# Temperature-Responsive Tensile Actuator Based on Multi-walled Carbon Nanotube Yarn

**DOI:** 10.1007/s40820-016-0084-6

**Published:** 2016-02-27

**Authors:** Hyunsoo Kim, Jae Ah Lee, Hyeon Jun Sim, Márcio D. Lima, Ray H. Baughman, Seon Jeong Kim

**Affiliations:** 1 Center for Self-powered Actuation and Department of Biomedical Engineering, Hanyang University, Seoul, 04763 South Korea; 2 The Alan G. MacDiarmid NanoTech Institute, University of Texas at Dallas, Richardson, TX 75083 USA

**Keywords:** Time–temperature, Actuator, Carbon nanotube, Paraffin, Coiled yarn, Dual-Archimedean

## Abstract

Many temperature indicators or sensors show color changes for materials used in food and medical fields. However, they are not helpful for a color-blind person or children who lack judgment. In this paper, we introduce simply fabricated and more useful low-temperature indicator (~30 °C) for devices that actuates using paraffin-infiltrated multi-walled carbon nanotube (MWCNT) coiled yarn. The density difference of MWCNT yarn provides large strain (~330 %) when heat causes the melted polymer to move. Furthermore, the MWCNT yarn decreases the melting point of paraffin. These properties allow control of the actuating temperature. In addition, mechanical strength was enhanced by MWCNT than previously reported temperature-responsive actuators based on shape memory polymers. This simply fabricated temperature indicator can be applied in latching devices for medical and biological fields.

**Electronic supplementary material:**

The online version of this article (doi:10.1007/s40820-016-0084-6) contains supplementary material, which is available to authorized users.

## Introduction

Time–temperature indicators have been studied historically as an important issue for ensuring the potency of life-saving vaccines around the world. Numerous companies have already developed them and now sell such products on the world markets. These indicators look like rectangular and flat laminates with layers of paper and film. They are used to monitor the cold chain for perishable food products, vaccines, blood, and many other applications. However, the sensor is typically placed inside boxes near the temperature-sensitive products and displays color changes by estimating the periods in which the products were exposed to above-threshold temperatures during transportation and storage. Recently, temperature-responsive indicators using chromatic materials have also been studied for other practical applications [1–3]. For example, an ink that can change color at high temperature was invented by adding polydiacetylene (PDA) to recognize counterfeit banknotes [4]. Gou et al. [2] showed color changes of a PDA/amphiphilic copolymer by exposure over a threshold temperature. Although these time–temperature indicators show easily recognized visual results, they are difficult to quantify the degree of temperature change and they cannot be used for some specialized application, which requires irreversible tensile work.

We focused on the more useful case of monitoring items that are necessary for life, such as food, vaccines, and blood, by mechanically actuating a signal when the product quality should be checked. Current approaches to biomedical devices such as those used in orthopedic surgery or cardiovascular applications, and which use shape memory thermoplastic polymers (SMPs), have shown recoverable strain; however, they also require high mechanical strength [5–7]. Lendlein et al. [8] introduced biodegradable, elastic SMPs, and interestingly described how they are used for shape memory sutures for wound closure. Shandas et al. [6] also suggested using stents with a high amount of perforation, generating a fast response and complete recovery. When the temperature reaches above the glass transition temperature (*T*_g_), the shape memory effect is actuated. However, yarn type actuator that elongates in one direction by environmental temperature change has not been reported.

Here, we demonstrate a temperature-responsive actuator in the temperature range between 35 and 45 °C using paraffin-infiltrated multi-walled carbon nanotube (MWCNT)-coiled yarn. As MWCNT yarn squeezes, the phase transited polymer, actuation occurred. Paraffin-infiltrated MWCNT-coiled yarns provided ~330 % tensile actuation as an elongation under a stress of 2.75 MPa. We also observed the tensile actuation behavior of the paraffin-infiltrated-coiled yarns according to various loads and exposure times at specific temperatures. Moreover, because MWCNT yarn has not only high electrical and thermal conductivity but also have high mechanical strength, this actuator has superior mechanical properties than previously reported SMP actuators. A possible application for the temperature-responsive MWCNT-based actuator is the latch device preventing lid opening for bottles containing vaccines, food, and blood when these are exposed to temperatures over the threshold for a certain period of time.

## Experimental

### Materials

The paraffin wax (melting temperature 53–57 °C) was purchased from Sigma-Aldrich. Spinnable MWCNT forests were grown on a Si wafer by the chemical vapor deposition method [9].

### Fabrication of Paraffin-Infiltrated MWCNT-Coiled Yarn

The uncoiled yarn was prepared by twisting four-, eight-, and twelve-layer MWCNT sheets with width and length of ~0.8 and 7.5 cm, respectively, in ethanol solution. A relatively low-inserted twist of about ~2,084 turns per meter was needed to induce high porosity and infiltration of paraffin during dipping. After twist insertion, both ends of the uncoiled yarn were fixed to a glass slide using carbon tape. The yarn, with both ends fixed, was dipped in melted paraffin for 10 s, and more twist was provided until the coiled structure was formed. A constant load of ~5 mN was applied to the paraffin-infiltrated MWCNT yarn when additional twist was provided.

### Experimental Setup

All tensile actuation measurements were conducted using a noncontact linear displacement sensor. The paraffin-infiltrated MWCNT-coiled yarn was tethered and supported a paddle with various loads (1, 5, and 10 mN) on the free end. The thermocouple was attached near the paraffin-infiltrated MWCNT-coiled yarn, covered with a glass tube (7 mm diameter), and a Ni/Cr (8:2) wire was wound on the glass tube to heat it up. The Ni/Cr wire was connected with an adjustable resistance (500 Ω, 5 kΩ) and a power supply, resulting in control of the heating rate for the ambient air temperature.

The differential scanning calorimetry (DSC) curve was obtained using Auto DSC-Q20 (TA Instruments). Thermogravimetric analysis (TGA) was measured with SDT Q600 (TA Instruments). The tensile strain was measured using a metal-target-sensing noncontact linear displacement sensor (LD 701) that was purchased from Omega. The heat source using Ni/Cr (8:2) alloy wire was purchased from Korea electronics market. A DC power supply (Protek 303Q) was used to apply current for heating of the Ni/Cr alloy wire. The ambient air temperature was measured during heating using a Type K thermocouple, PFA insulation, 40 AWG, 36-inch length (part number 5SC-TT-K-40-36) from Omega. The surface morphology of paraffin-infiltrated MWCNT-coiled yarn before and after heating was recorded with an SEM (Hitachi S4700, Japan).

## Results and Discussion

**Fig. 1:**
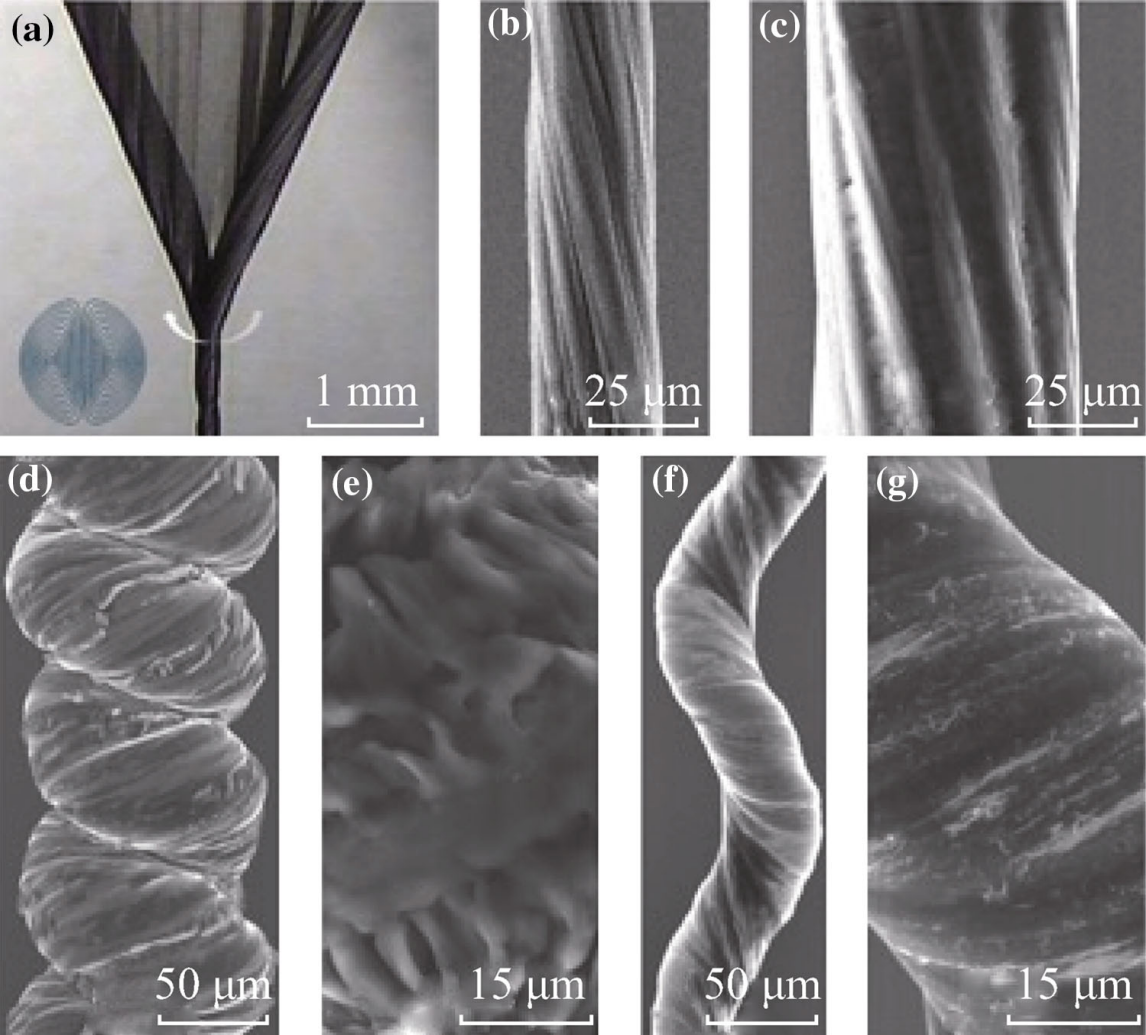
**a** Optical microscopy image of spinning, showing wedge edges being twisted to form a dual-Archimedean scroll yarn. The* inset* shows schematic dual-Archimedean cross section. SEM images. **b** Densified neat single MWCNT yarn. **c** Paraffin-infiltrated MWCNT yarn. **d**, **f** Paraffin-infiltrated MWCNT-coiled yarn before and after heating, respectively. **e**, **g** Magnified SEM images of paraffin-infiltrated MWCNT-coiled yarn before and after heating, respectively

Figure 1a presents an optical microscope image of a neat four-layer-stacked MWCNT sheet that was twisted into a yarn. The opposite lateral sides of the wedge structure twisted into yarn during inserting to produce gradually dual-Archimedean scrolls, as shown in Fig. 1a (inset). During the twisting, the density of yarn gradually increased, and finally an MWCNT yarn with different densities along the radial direction was fabricated. Figure 1b, c shows scanning electron microscope (SEM) images of a neat MWCNT and a paraffin-infiltrated MWCNT yarn structure, respectively. The neat MWCNT yarn has a high porosity of ~69 %, resulting in 310 ± 25 wt% paraffin loading. From the SEM measurement, the diameter and the bias angle of neat yarn were ~30.6 μm and ~11.5°, respectively; the diameter of paraffin-infiltrated MWCNT yarn was ~64.4 μm, which was ~2.1 times thicker than the neat yarn and the bias angle was ~12.5°. After paraffin infiltration in the neat MWCNT yarn, more twisting was provided until a fully coiled structure was formed (Fig. 1d). A surface image of the paraffin-infiltrated MWCNT-coiled yarn for a tensile actuator shows thick and rough paraffin covering the overall surface of the coiled yarn (Fig. 1e). The thin and elongated paraffin-infiltrated MWCNT-coiled yarn after heating to ~80 °C under a stress of 1.42 MPa is shown at two magnifications in Fig. 1f, g. The diameter dramatically decreased from ~67.5 μm before heating to ~39.5 μm after heating.

**Fig. 2:**
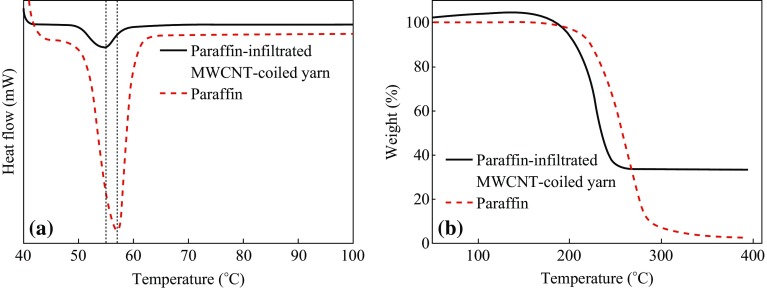
**a** DSC thermogram of paraffin-infiltrated MWCNT-coiled yarns and pure paraffin with a heating rate of 10 °C min^−1^ under a flow of 100 ml min^−1^ in air. Each *dotted lines* represent endothermic peak. **b** TGA curve of a paraffin-infiltrated MWCNT-coiled yarn and pure paraffin

The results of differential scanning calorimetry (DSC) of the paraffin-infiltrated coiled yarn (Fig. 2a) exhibited an endothermic peak at ~55 °C. The temperature of endothermic peak was decreased from ~57 to ~55 °C as compared with pure paraffin. In DSC thermograms, the dominant peaks represent the solid–liquid (melting) phase change. According to the literature [10], the melting point of paraffin increases with increasing the number of carbon atoms in the paraffin chain. This result implies that the temperature-responsive actuator has a potential application in responding at the various temperatures when paraffin with a different melting point is used as a guest material. The measurements were conducted by increasing the temperature from room temperature to 400 °C at a rate of 10 °C min^−1^ under a flow of 100 ml min^−1^ in air. Thermogravimetric analysis (TGA) showed the loss of paraffin wax during heating (Fig. 2b). Pure paraffin showed significant weight decrease after ~210 °C. On the other hand, weight decrease of the paraffin-infiltrated coiled yarn occurred around ~200 °C by evaporation of the paraffin wax. The temperature range of the TGA measurement was from room temperature to 400 °C, at a rate of ~10 °C min^−1^ in air. These results show thermal behaviors of the paraffin changed after being infiltrated into the MWCNT yarn. Moreover, as shown in TGA curve (Fig. 2b) of paraffin-infiltrated MWCNT-coiled yarn, ~40 wt% MWCNT is remained above ~400 °C, which means that this actuator has high thermal stability.

**Fig. 3:**
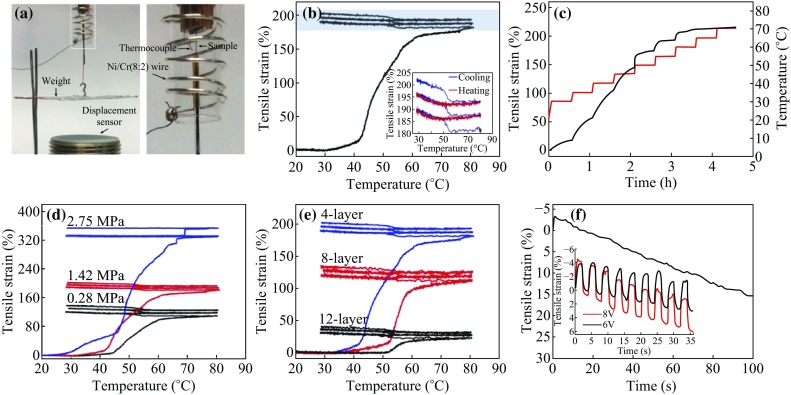
**a** Experimental setting of thermal actuation. The *right* photograph is enlarged from the *dotted box*. **b** Tensile strain as a function of ambient air temperature for three cycles of a paraffin-infiltrated four-layer MWCNT-coiled yarn with a stress of 1.42 MPa. The *inset* shows an enlarged tensile strain in the *blue box*. **c** Tensile strain of a paraffin-infiltrated MWCNT-coiled yarn versus exposure time at certain temperatures. Each temperature was maintained for 28 min and was increased by 5° from 30 to 70 °C. **d** Dependence of the tensile strain on the loads. **e** Dependence of the tensile strain on the number of MWCNT layers before being twisted to make yarn. **f** Time dependence of the tensile strain for an 8 V square-wave pulse that was 100 s long. The *inset* shows tensile stain versus time for paraffin-infiltrated four-layer MWCNT-coiled yarn when the 6 V (*black*) and 8 V (*red*) with 0.25 Hz square-wave voltage with 50 % duty cycle was applied

For measuring the mechanical properties, all tensile actuation was obtained from a noncontact linear displacement sensor that collects data from vertical movements of the flat weight located at the end of the tensile actuator. The paraffin-infiltrated MWCNT-coiled yarn was slowly heated by Ni/Cr (8:2) alloy wire using adjustable resistance and DC power supply. An experimental setup is shown in Fig. 3a, and detailed experimental process is described in the Experimental Section. The tensile actuation results of the muscular structure of the paraffin-infiltrated MWCNT-coiled yarn (Fig. 1d) are shown in Fig. 3b and Movie S1. The large tensile elongation of ~180 % was obtained at an ambient air temperature of between 40 and 60 °C in the first heating. The rate to initially large elongation started at ~42 °C by 50 % is 14.2 % (°C)^−1^ and the elongation is asymptotically approached. The elongated coiled yarn was subjected to two more cycles of cooling and heating with a load of 1.42 MPa. The tensile actuations during the second and third cycles were also elongated; however, the stroke was much smaller than in the first cycles. The first ~5 % contraction during the overall heating and ~10 % elongation during cooling at ~55 °C were obtained in the second and third cycles because of rearrangement and volume change of the polymer in the MWCNT-coiled yarn during heating and cooling. The first tensile actuation started ~10 °C below the melting temperature of the paraffin wax used. The reason of such a large tensile elongation at the first heating cycle could be explained by the rearrangement of paraffin wax in the MWCNT-coiled yarn. When the paraffin wax in the yarn was exposed close to the melting temperature (~55 °C), the melted paraffin wax flowed out and ran along the yarn. The tensile elongation was accelerated by a load suspended from the end of a coiled yarn by compression.

Figure 3c shows the tensile actuation of the paraffin-infiltrated MWCNT-coiled yarn vs exposure time at certain temperatures, with increments of 5°C from 30 to 70 °C. Each temperature was maintained for 28 min. From these measurements, the first elongation of ~15 % started at ~30 °C, and the major elongation (over 30 % at each temperature) occurred between 35 and 45 °C. The elongation was asymptotically approached at each temperatures and almost finished at ~60 °C. The final tensile elongation of this sample was ~214 %, which is similar to that in Fig. 3b. The applied load from 0.28 to 2.75 MPa generated tensile elongation of 109–330 % (Fig. 3d). Also, the elongation starting points of temperature gradually shifted. Figure 3e shows that the tensile actuation of paraffin-infiltrated MWCNT-coiled yarn consisted of a layer of various numbers of MWCNT sheets under the same weight (~5 mN) during heating. The total tensile actuation of coiled yarns twisted by four-, eight-, and twelve-layer MWCNT sheets was obtained as 180, 112, and 28 % at ~80 °C, respectively. The actuation starting points became faster with decreasing the number of MWCNT sheets in the layer. Tensile strain of our yarn actuator is highly related to weight percent of MWCNT because the MWCNT layer acts as barrier to prevent guest paraffin from flowing out when heated. Therefore, when less MWCNT layers, the guest paraffin flows out more easily and the yarn actuator shows higher tensile performance.

We also checked the tensile actuation of a paraffin-infiltrated MWCNT-coiled yarn powered by electrical heat. When the hybrid yarn was exposed to heat for 100 s at an 8 V square-wave pulse, a contraction of 3.3 % was generated at first for a short time and then steadily elongation occurred during heating (Fig. 3f). The tensile strains of −3.7 and −4.2 % were obtained by applying 0.25 Hz, 6 V, and an 8 V square-wave voltage at 50 % duty cycle to lift a load that provided a 3.9 MPa stress as shown in Fig. 3f (inset). The hybrid-coiled yarn experienced creep during the cycles and the creep constantly increased at higher applied voltage. From this experiment, the paraffin volume expanded during heating, and simultaneously, paraffin slowly flowed out and ran along the yarn when the paraffin was exposed close to its melting point. We measured the weight of paraffin-infiltrated MWCNT-coiled yarn before and after heating by oven. An average weight decrease of ~20.6 % occurred during heating. Such a large change in weight makes a huge volume change in the yarn, with the result of high elongation of the coiled yarn close to the melting point during heating.

**Fig. 4:**
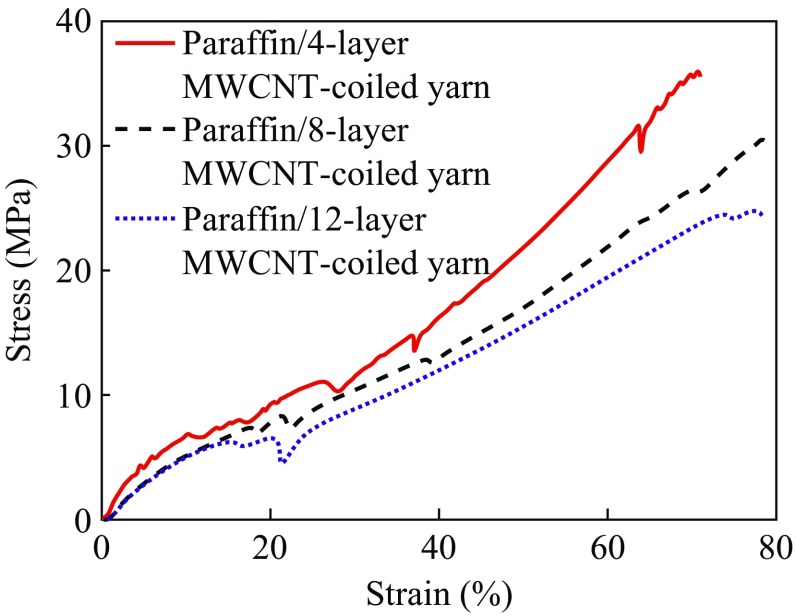
Stress–strain curve for paraffin-infiltrated MWCNT-coiled yarns twisted in four- eight-, and twelve-layer MWCNT sheets at room temperature

We performed tensile tests of paraffin-infiltrated MWCNT-coiled yarn, consisting of a varying number of MWCNT sheet layers at room temperature (Fig. 4). The specific mechanical strength and modulus of paraffin-infiltrated four-layer MWCNT-coiled yarn were 38 ± 4 and 132 ± 18.6 MPa, respectively. In the case of paraffin-infiltrated eight-layer MWCNT-coiled yarn, the specific mechanical strength and modulus were 35 ± 5 and 112.7 ± 28.2 MPa, respectively. Finally, the specific mechanical strength and modulus of paraffin-infiltrated twelve-layer MWCNT-coiled yarn were 29 ± 4 and 104.3 ± 3.3 MPa, respectively. This mechanical strength was ~10 times higher than that of polydiolcitrate polyester elastomers with shape memory properties [11]. Moreover, the strength of the paraffin-infiltrated MWCNT-coiled yarn exceeded other SMP and SMP/CNT composites that require high-strength structural components for specific medical and biological applications such as stents or wound closure (ultimate strength of up to about 4-30 MPa) [12–14]. The strains of the paraffin-infiltrated with four-, eight-, and twelve-layer MWCNT-coiled yarn were 70, 79, and 78 %, respectively.

## Conclusion

In conclusion, we developed a temperature-responsive high-strain paraffin-infiltrated MWCNT-coiled yarn actuator by infiltration method using aligned MWCNT sheets that have high strength, thermal, and electrical conductivity. Because the temperature-responsive actuator responds directly, it is more practical and useful than chromatic sensors and indicators of paper or film types. The demonstrated tensile elongation generated ~330 % under a stress of 2.75 MPa when this actuator was exposed to near its melting temperature due to melted paraffin flow out from inner of the MWCNT-coiled yarn by physical force. The specific mechanical strength and modulus of paraffin-infiltrated four-layer MWCNT-coiled yarn (38 ± 4 and 132 ± 18.6 MPa, respectively) were much improved over the mechanical strength and modulus of the SMP actuator, which requires enhanced mechanical properties. In contrast with previous chromatic indicators that just displaying color change, the low-temperature operation, high strain, and mechanical properties suggest not only application in a latching device for vaccine and food but also in medical and biological devices.

## Electronic supplementary material

Below is the link to the electronic supplementary material.

Supplementary material 1 (AVI 7050 kb)
